# Mental health risks in undergraduate students with body image dissatisfaction

**DOI:** 10.1192/j.eurpsy.2026.11237

**Published:** 2026-07-31

**Authors:** E. L. Nikolaev, I. E. Poverinov, E. Y. Vasilyeva, S. A. Petunova, A. N. Zakharova, D. V. Hartfelder, E. Y. Lazareva, N. L. Maksimova, E. M. Litvinova, G. S. Dulina, N. V. Grigorieva, H. Kumar

**Affiliations:** 1 Department of Social and Clinical Psychology; 2 Department of Philosophy, Sociology and Pedagogy; 3 Medical Faculty, I.N. Ulianov Chuvash State University, Cheboksary, Russian Federation

## Abstract

**Introduction:**

Body image dissatisfaction and incorrect body image perceptions are widely spread among students (Divecha et al., 2022). More often body image dissatisfaction is associated with disordered eating and eating disorders, as well as with social anxiety symptoms.

**Objectives:**

Our objective was to find out if an incorrect perception of a student’s body image could be accompanied by more significant signs of mental disorders or high risks of their further disorders.

**Methods:**

Our objective was to find out if an incorrect perception of a student’s body image could be accompanied by more significant signs of mental disorders or high risks of their further disorders.

**Results:**

The findings revealed that every fourth student (26.4%) had body image dissatisfaction, although their mean BMI value did not exceed the norm – 21.7±4.2. The females were dissatisfied with their body image reliably more often than the males (p<.05). All the SCL-90-r indicators in the students with body image dissatisfaction were reliably higher (p<.05) than those in the students, who did not have such dissatisfaction. They also reliably exceeded the standard showings according to this technique, including Global Severity Index, Positive Symptom Distress Index and Positive Symptoms Total (p<.05). We found maximal differences on the scales of Paranoid Ideation, Hostility, Somatization and Psychoticism. As for concomitant symptoms, most frequent were morning insomnia, difficulty falling asleep, bad appetite, and overeating. Although these findings do not testify to the presence of objective mental health problems in students with body image dissatisfaction, they allow assuming that in the perspective they may have high risks of further mental health disorders.

**Conclusions:**

No doubt, this group of students should receive more attention from mental health professionals. Prevention of mental health risks in students with body image dissatisfaction should focus on the prevention of negative body images, disordered eating and eating disorders, as well as teaching stress management and motivation for healthy physical activity. University health education programs may also improve body image and prevent eating disorders in university students of both sexes.

**Disclosure of Interest:**

None Declared

